# Research progress in augmentation strategies for PD-1/PD-L1 inhibitors in bladder cancer: from biological determinants to clinical applications

**DOI:** 10.3389/fimmu.2026.1802404

**Published:** 2026-04-17

**Authors:** Yaxin Cheng, Wenzhi Deng, Yunqing Liu, Guanjun Chen, Ke Cao

**Affiliations:** 1 Department of Oncology, Third Xiangya Hospital of Central South University, Hunan, Changsha, China; 2 Department of Pathology, Third Xiangya Hospital of Central South University, Hunan, Changsha, China

**Keywords:** biomarkers, bladder cancer, combination strategies, PD-1/PD-L1 inhibitors, tumor microenvironment

## Abstract

PD-1/PD-L1 immune checkpoint inhibitors (ICIs) have demonstrated significant clinical efficacy in the treatment of bladder cancer. However, heterogeneous patient responses continue to limit the widespread adoption and overall effectiveness of these therapies. Consequently, identifying strategies to enhance treatment response has become a primary focus of current oncological research. This review summarizes the biological determinants of immune response in bladder cancer, including sex, gut microbiota, molecular subtypes, and the tumor microenvironment (TME). Furthermore, we evaluate key predictive biomarkers for ICI response, such as PD-L1 expression, tumor mutational burden (TMB), and circulating tumor DNA (ctDNA). Synergistic combination strategies—incorporating chemotherapy, radiotherapy, targeted therapy, and nanomedicine—are also detailed to provide novel insights into bladder cancer immunotherapy. Ultimately, the systematic elucidation of immune response mechanisms combined with technological innovation will facilitate the optimization of therapeutic strategies, leading to improved clinical outcomes for patients.

## 1 Introduction

Globally, bladder cancer ranks as the tenth most prevalent malignancy and the second most common cancer of the urinary tract. Due to its high incidence, significant risk of recurrence, and therapeutic resistance, it has emerged as a major global public health concern (1–3). Approximately 95% of cases are identified as urothelial carcinoma, which is further categorized into non-muscle-invasive bladder cancer (NMIBC) and muscle-invasive bladder cancer (MIBC). Although conventional modalities—including surgery, radiotherapy, and chemotherapy—have achieved clinical success, they often fail to fundamentally eradicate the risks of systemic metastasis and local recurrence (4).

Recently, anti-tumor therapies based on PD-1/PD-L1 immune checkpoint inhibitors (ICIs) have demonstrated substantial progress. ICIs function by disrupting immune checkpoint protein signaling, thereby counteracting tumor immune evasion mechanisms and restoring the capacity of the immune system to recognize and eliminate malignant cells. In the context of bladder cancer, PD-1/PD-L1 inhibitors are the most extensively utilized ICIs; they specifically target the PD-1/PD-L1 axis to alleviate T-cell suppression and reactivate anti-tumor immunity (5). Current PD-1 inhibitors include nivolumab, pembrolizumab, cemiplimab, and dostarlimab, while the PD-L1 class encompasses atezolizumab, durvalumab, and avelumab.

PD-1/PD-L1 inhibitors have achieved substantial clinical milestones in the management of bladder cancer. Following the landmark JAVELIN Bladder 100 trial, avelumab has been established as the standard-of-care first-line maintenance therapy for patients with locally advanced or metastatic urothelial carcinoma (mUC) who did not progress after 4–6 cycles of first-line platinum-based chemotherapy (6). Crucially, this maintenance benefit is observed across clinical subgroups, regardless of initial cisplatin eligibility or whether patients received cisplatin- or carboplatin-based induction regimens. For patients who are cisplatin-ineligible, pembrolizumab remains an option for first-line monotherapy in PD-L1-positive mUC (KEYNOTE-052) (7, 8). Additionally, nivolumab received approval for second-line treatment of platinum-resistant mUC based on the CheckMate 275 trial (9). The key clinical trial data of these FDA-approved PD-1/PD-L1 inhibitors are summarized in **Table 1**. Furthermore, the treatment landscape has recently undergone a paradigm shift with the emergence of combination therapies, such as enfortumab vedotin plus pembrolizumab (EV-302) and nivolumab plus gemcitabine-cisplatin (CheckMate 901), which have demonstrated superior outcomes in the first-line setting (10, 11).

**Table 1:** Clinical trial data of FDA-approved PD-1/PD-L1 inhibitors for the treatment of bladder cancer.

Medication	Trial	Treatment group	Phase	Patients	Disease stage	Line of therapy	Characteristics
Atezolizumab	IMvigor210	Atezolizumab monotherapy who are ineligible for cisplatin-containing chemotherapy	II	123	mMIBC	1st	ORR:23% mPFS:2.7mo mOS:15.9mo
	IMvigor130	Atezolizumab+ platinum-based chemo (A) vs. Atezolizumab monotherapy (B) vs. platinum-based chemo (C)	III	1213	mMIBC	1st	mPFS: 8.2 vs. 6.3mo (A vs. C) mOS: 16 vs. 13.4mo (A vs. C) mOS: 15.7 vs. 13.1mo (B vs. C)
Durvalumab	NCT01693562	Patients treated with durvalumab	I/II	61	mMIBC	2nd	ORR:31% mOS: 14.1 mo
	NIAGARA	Patients with MIBC who had not received previous systemic therapy for bladder cancer and were eligible for radical cystectomy	III	1063	MIBC	1st	EFS: 67.8% vs. 59.8% (duvalumab group vs. control group) OS :82.2% vs. 75.2% (duvalumab group vs. control group)
Avelumab	JAVELIN Bladder 100	Avelumab (A) maintenance + BSC vs. BSC alone (B) in patients without disease progression after 1L platinum-based chemo	III	700	mMIBC	1st	mOS: 21.4 vs. 14.3 mo (A vs. B) mOS: not estimable vs. 17.1 mo (A vs. B)
Pembrolizumab	keynote045	Pembrolizumab (P) vs. chemo-regimen (docetaxel or paclitaxel or vinflunine) (C)	III	542	mMIBC	2nd	mOS: 10.3 vs. 7.4mo (P vs C) mOS: 8.0 vs. 5.2mo (P vs C, PD-L1 status CPS ≥ 10%)
	keynote052	patients with pathologically confirmed bladder cancer who had not received previous chemotherapy and were not candidates for cisplatin-based therapy	II	370	mMIBC	1st	mOS :11.3mo, 4yearOS:20%, patients with CPS≥10 have better OS
Nivolumab	Checkmate275	Nivolumab after platinum-based chemo	I/II	270	mMIBC	2nd	ORRs in the low, medium, and high TMB groups :13.0%vs.19.6%vs.31.9%, it was found that absence of PD-L1 expression and low TMB levels did not appear to benefit from nivolumab treatment.
EV+ Pembrolizumab	EV-302	Enfortumab Vedotin + Pembrolizumab (P+EV) vs. Platinum-based chemotherapy (C)	III	886	mMIBC	1st	mOS: 31.5 vs. 16.1 mo (P+EV vs. C)
Nivolumab + Gem/Cis	CheckMate 901	Nivolumab (N) + Gemcitabine-Cisplatin (G+C) vs. Gemcitabine-Cisplatin (G+C)	III	608	mMIBC	1st	mOS: 21.7 vs. 18.9 mo (N+G+C vs. G+C)

MIBC, muscle-invasive bladder cancer; mMIBC, metastatic muscle-invasive bladder cancer; ORR, objective response rate; mPFS, median progression-free survival; mo, months; mOS, median overall survival.

Despite these advancements, patient response to PD-1/PD-L1 inhibitors remains highly heterogeneous. The overall objective response rate (ORR) for monotherapy typically hovers around 20%, frequently challenged by primary or secondary resistance. Consequently, identifying strategies to augment therapeutic response is a central focus of contemporary research.

Recent studies have identified the loss of the Y chromosome (LOY)—a frequent occurrence in aging men—as a unique biological paradox (12, 13). While LOY confers a significant growth advantage and high invasiveness by driving metabolic remodeling via the DDR2 pathway (notably through activated aerobic glycolysis) (14, 15), it simultaneously induces a state of profound exhaustion in CD8^+^ T cells (12). This extreme immune evasion mechanism essentially strips the tumor of its “immune camouflage,” rendering it highly vulnerable to PD-1/PD-L1 blockade. This “high-aggression, high-sensitivity” phenotype elucidates the molecular logic of LOY in cancer progression and provides a critical framework for understanding immune heterogeneity and identifying patients most likely to benefit from therapy (16).

This review summarizes recent progress in enhancing PD-1/PD-L1 inhibitor efficacy in bladder cancer. We systematically analyze the biological determinants of immune response, evaluate multidimensional biomarkers for predicting efficacy, and discuss emerging combination strategies designed to achieve therapeutic synergism.

## 2 Biological determinants of immune response in bladder cancer

### 2.1 Sexual dimorphism

Distinct differences exist in the composition and function of the immune system between sexes, with females generally exhibiting higher baseline immune activity than males. Research by Kwon et al. elucidated the mechanisms underlying sex bias in bladder cancer, demonstrating that androgen receptor (AR) signaling promotes CD8^+^ T-cell exhaustion via TCF1, thereby mediating increased susceptibility in males (17). Furthermore, CD8^+^ T cells in males exhibit diminished anti-tumor effector functions and reduced stemness compared to those in females (18).

Mechanistically, AR suppresses the activity and stem-like properties of CD8^+^ T cells in malignancies by modulating epigenetic and transcriptional differentiation. Consequently, androgen deprivation or the pharmacological blockade of AR signaling has been shown to enhance the clinical efficacy of anti-PD-1 therapy (17, 19).

The Y chromosome is indispensable for male sex determination and spermatogenesis. In aging males, the mosaic LOY in hematopoietic cells is closely associated with oncogenesis (20–22). Within bladder cancer cohorts, the prevalence of LOY ranges from 10% to 40% (4, 22, 23). Recent evidence suggests that LOY—along with the loss of Y-linked chromatin-modifying genes such as KDM5D and UTY—facilitates bladder cancer progression through immune evasion (24). Conversely, other studies indicate that LOY-mutant cancer cells modulate T-cell function to promote a state of exhaustion, which paradoxically sensitizes the tumor to PD-1-targeted immunotherapy (12).

Given the profound impact of sexual dimorphism on ICI response, future clinical trials must ensure balanced sex representation. Furthermore, integrating sex-based biological factors into the design of combination therapeutic regimens is essential for optimizing bladder cancer treatment.

### 2.2 Gut microbiota

The gut-bladder axis has emerged as a critical determinant of immunotherapy success, with the intestinal microbiome functioning as a systemic “rheostat” that modulates host anti-tumor immunity. However, as the field transitions from mechanistic exploration to clinical application, it is essential to rigorously distinguish high-level clinical evidence—such as established negative prognostic factors—from early, hypothesis-generating exploratory concepts.

#### 2.2.1 Antibiotic-induced dysbiosis: a validated negative prognostic factor

Antibiotic exposure is now recognized as a definitive negative prognostic factor influencing the efficacy of ICIs in bladder cancer, supported by extensive clinical outcome data. A systematic review and meta-analysis of 13 studies involving 5,095 patients with urothelial carcinoma provided Level II evidence linking antibiotic use to significantly poorer survival (25). Specifically, the pooled hazard ratio (HR) for overall survival (OS) was 1.45 (95% CI 1.25–1.68), while the pooled HR for progression-free survival (PFS) was 1.40 (95% CI 1.05–1.87).

The timing of exposure is critical; reductions in OS, PFS, and ORR are most pronounced when antibiotics are administered within three months before or after the initiation of ICI therapy (26).

Fidelle et al. (27) elucidated the underlying mechanism, demonstrating that antibiotic-induced dysbiosis leads to the over-colonization of Enterocloster species. This microbial shift results in the downregulation of Mucosal Addressin Cell Adhesion Molecule-1 (MAdCAM-1), a gut checkpoint molecule. When MAdCAM-1 expression is disrupted, tumor immune evasion is promoted, thereby compromising the therapeutic efficacy of ICIs.

#### 2.2.2 Microbiome modulation: exploratory concepts and early-phase clinical trials

In contrast to the well-documented detrimental effects of antibiotics, strategies designed to “augment” the microbiota remain largely investigative or hypothesis-generating.

Preclinical Mechanisms and Metabolites: Utilizing murine models of bladder cancer, Mager et al. (28) provided high-quality hypothesis-generating data demonstrating that specific commensals, such as Bifidobacterium pseudolongum, significantly enhance ICI efficacy. This synergism is mediated by the production of the metabolite inosine, which promotes T-cell activation. These findings identify a potential therapeutic target for future interventions.

Probiotic Interventions: The butyrate-producing probiotic CBM588 (Clostridium butyricum) has shown preliminary positive signals in genitourinary malignancies. In a retrospective cohort study of 44 patients with advanced urothelial carcinoma, the combination of CBM588 and pembrolizumab improved median PFS to 10.2 months, compared to 3.9 months in the control group (HR 0.074) (29). However, due to its retrospective nature, this result is currently classified as a hypothesis-generating observation. Prospective validation is ongoing in trials such as IMPROVE (NCT06904573).

Fecal Microbiota Transplantation (FMT): FMT aims to fundamentally reshape the host’s intestinal ecosystem through the transplantation of whole microbial communities from healthy donors or ICI responders. In melanoma and renal cell carcinoma (RCC), FMT has demonstrated substantial potential to reverse primary resistance to ICIs. For instance, the Phase IIa TACITO trial in metastatic RCC patients showed that FMT from ICI-responsive donors, combined with pembrolizumab, significantly increased the 12-month PFS rate (70% vs. 41%) and achieved a median PFS of 24 months (30).

In bladder cancer, however, clinical evidence for FMT remains sparse, consisting primarily of case reports and early-stage pilot explorations. While individual cases indicate that FMT can successfully resolve ICI-induced severe colitis—accompanied by successful donor strain engraftment—FMT has not yet been validated as a means to enhance ICI response in large-scale, prospective randomized controlled trials (31). Consequently, FMT in the context of bladder cancer remains in an early experimental stage. Key barriers to broad clinical implementation include safety concerns, the lack of standardized donor screening criteria, and the determination of optimal transplantation frequency.

### 2.3 Molecular subtyping

Bladder cancer is defined by extensive molecular and clinical heterogeneity, which has led to the development of various classification systems, including the TCGA, UNC, MDA, Lund, and Baylor frameworks (32–36). Despite these efforts, these subtyping models often fail to provide consistent predictions regarding the efficacy of ICIs. For instance, while the IMvigor210 trial identified the TCGA Luminal Cluster II as the subtype most responsive to atezolizumab (34% ORR), the CheckMate 275 trial found that the Basal Cluster III subtype exhibited the highest response rate to nivolumab (30%) (9, 37). These conflicting observations suggest that static molecular labels may fail to capture the dynamic functional states of the tumor microenvironment (TME). Consequently, the functional phenotype of the TME, rather than mere taxonomic classification, appears to be the primary driver of differential ICI sensitivity.

In luminal tumors, particularly the luminal papillary subtype, frequent activating mutations in FGFR3 drive oncogenic progression while fostering a “cold” or “non-T-cell-inflamed” immune landscape (38, 39). This activation facilitates the ubiquitinated degradation of PD-L1 via the phosphorylation of the E3 ubiquitin ligase NEDD4 (40). Furthermore, FGFR3 signaling induces inflammatory fibroblasts (iCAFs) to secrete factors such as CXCL12, which physically impede T-cell recruitment and infiltration, resulting in an “immune-excluded” phenotype (41). Patients with these profiles often demonstrate intrinsic resistance to ICI monotherapy, frequently showing poorer OS than those with FGFR3 wild-type tumors (42). Addressing these cases may require the strategic use of FGFR inhibitors to neutralize immunosuppressive signaling, thereby “warming” the microenvironment and overcoming the immune-exclusion barrier (43, 44).

Conversely, basal/squamous (Ba/Sq) tumors typically present as “hot” or “T-cell-inflamed” tumors characterized by high CD8^+^ T-cell infiltration but accompanied by robust adaptive immune resistance (38). These tumors frequently overexpress multiple inhibitory molecules, including PD-1, PD-L1, CTLA-4, LAG-3, and TIM-3, which collectively drive infiltrating T cells into a state of profound exhaustion (45–47). In such a deeply immunosuppressive environment, blocking the PD-1/PD-L1 axis alone is often insufficient to restore T-cell function, as the tumor may utilize compensatory pathways to maintain immune evasion (48). Therefore, more aggressive strategies, such as the dual blockade of PD-1 and CTLA-4, are necessary to prime T-cell activity and reverse exhaustion (49, 50). Ultimately, shifting the clinical focus from traditional molecular subtyping to the specific functional phenotypes of the TME provides a rational framework for interpreting trial outcomes and advancing individualized precision immunotherapy (44, 51).

### 2.4 Tumor microenvironment

**Figure 1:**
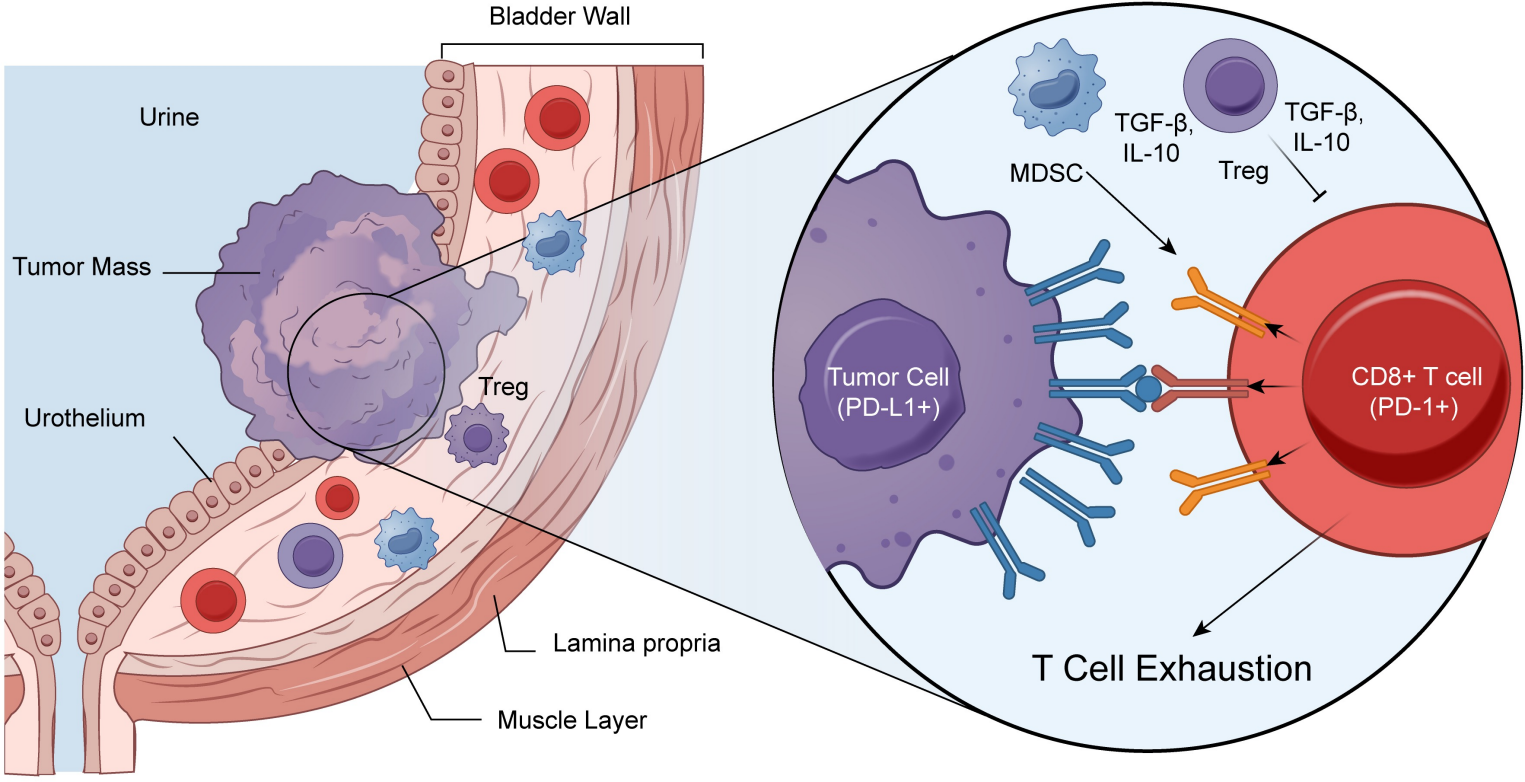
The immunosuppressive tumor microenvironment (TME) of bladder cancer and mechanisms of PD-1/PD-L1-mediated T cell exhaustion. A schematic illustration depicting the histological context and molecular interactions within the bladder cancer TME that lead to immune escape. (Left Panel) A cross-section of the bladder wall showing the anatomical layers: urothelium, lamina propria, and muscle layer, adjacent to the urine-filled lumen. An invasive tumor mass is shown developing from the urothelium. The TME is infiltrated by various immune cells, including effector CD8+ T cells, but is dominated by immunosuppressive populations such as regulatory T cells (Tregs) and myeloid-derived suppressor cells (MDSCs). (Right Panel) A magnified view highlighting the critical molecular synapse driving immune suppression. Tumor cells highly express Programmed Death-Ligand 1 (PD-L1), which engages with Programmed Death-1 (PD-1) receptors on the surface of infiltrating CD8+ T cells. This binding initiates inhibitory signaling cascades that result in T cell dysfunction and exhaustion, abrogating anti-tumor immunity. Concurrently, stromal cells like MDSCs and Tregs contribute to the hostile TME by secreting immunosuppressive cytokines, such as transforming growth factor-beta (TGF-β) and interleukin-10 (IL-10), further inhibiting effector T cell activity.

The TME serves as a critical niche for the growth, proliferation, and metastasis of malignant cells. It functions as a complex ecosystem integrated by diverse cellular lineages, signaling cytokines, the extracellular matrix (ECM), and metabolic intermediates. The intricate crosstalk among these heterogeneous constituents collectively modulates oncogenesis and disease progression (52), while fundamentally determining the therapeutic efficacy and clinical responsiveness of ICIs (**Figure 1**).

#### 2.4.1 Immune cell infiltration

Tumor-infiltrating lymphocytes (TILs) represent a diverse population of immune-active cells residing within the TME. Among these, CD8+ T cells—also known as cytotoxic T lymphocytes (CTLs)—are the primary effectors of anti-tumor immunity, capable of directly recognizing and lysing malignant cells (53). These cells secrete several critical cytokines, including interferon-γ (IFNγ) and tumor necrosis factor-α (TNFα). While IFNγ enhances tumor immunogenicity by upregulating the expression of MHC-I molecules on the cell surface, TNFα exerts direct cytotoxic effects and facilitates the recruitment of auxiliary immune cells, such as macrophages and natural killer (NK) cells, into the TME. These processes synergistically amplify the therapeutic efficacy of ICIs.

Based on the spatial distribution and infiltration patterns of T cells, tumors are categorized into three distinct phenotypes: immune-inflamed, immune-excluded, and immune-desert (54). The immune-inflamed phenotype is characterized by the robust infiltration of CD8+ T cells into the tumor parenchyma. In contrast, the immune-excluded phenotype exhibits high CD8+ T cell density that is primarily restricted to the tumor periphery or stroma. The immune-desert phenotype is defined by a marked paucity of CD8+ T cells within the tumor (55). Clinical evidence from Mariathasan et al. indicates that patients with immune-inflamed bladder cancer achieve the highest response rates to atezolizumab (56). Conversely, regulatory T cells (Tregs) exert immunosuppressive functions that inhibit the activity of CD8+ T cells and other immune effectors, thereby promoting tumor progression and immune evasion. For instance, Tregs suppress CD8+ T cell function through the secretion of inhibitory cytokines, including IL-10 (57).

Tumor-associated macrophages (TAMs) constitute the most abundant immune cell population within the TME. A preclinical study using a triple-knockout (Trp53, Pten, and Rb1) mouse model of bladder cancer demonstrated that responders to PD-1 inhibition harbored significantly higher macrophage counts than non-responders (58). TAMs typically adopt two divergent polarization states: M1 and M2. M1 macrophages maintain anti-tumor activity by activating immune responses and expressing high levels of TNFα, inducible nitric oxide synthase (iNOS), and IL-12. Conversely, M2 macrophages promote tumor growth and are characterized by the expression of CD163, CD206, arginase 1 (ARG1), and IL-10 (59, 60). Furthermore, Sun et al. reported that TAM polarization is closely linked to anti-PD-L1 response in the IMvigor210 trial, with M1-dominant tumors exhibiting greater sensitivity to atezolizumab (61). Consequently, while TAM density and polarization correlate with ICI efficacy, the precise underlying mechanisms remain to be fully elucidated.

Beyond the aforementioned populations, other components such as dendritic cells (DCs), NK cells, cancer-associated fibroblasts (CAFs) (62), and eosinophils also influence the responsiveness to ICI therapy. However, research specifically addressing these elements in the context of bladder cancer remains insufficient, necessitating more rigorous investigation.

#### 2.4.2 Non-cellular components

Tertiary lymphoid structures (TLS) are ectopic lymphoid aggregates that form in non-lymphoid tissues under chronic inflammatory or pathological conditions (63). Mature TLS typically consist of B-cell zones containing germinal centers, which are encapsulated by T-cell-rich regions. As critical hubs for tumor-immune interactions, the density of TLS correlates with robust adaptive immune cell infiltration and improved clinical outcomes across various malignancies, including bladder cancer (64, 65). In a study by Gao et al., patients receiving combination therapy with tremelimumab and durvalumab demonstrated higher response rates if they exhibited elevated baseline TLS density (66). Furthermore, high pre-treatment TLS density was associated with prolonged OS and recurrence-free survival (RFS) (66).

In contrast, data from the NABUCCO clinical trial—where patients received neoadjuvant PD-1 and CTLA-4 blockade—indicated no significant statistical correlation between baseline TLS levels and clinical response rates. However, a marked increase in TLS density was observed post-intervention in all cases achieving pathological complete response (67). These findings suggest that ICIs may enhance the local anti-TME by activating or recruiting TLS. Consequently, elucidating the role of TLS within the immunotherapy regulatory network is essential for developing personalized predictive models and biomarker-driven strategies for patient stratification.

Within the TME, chemokines and cytokines function as central hubs of the local immune response by synergistically regulating the activation, chemotactic migration, and phenotypic polarization of immune effectors (68). Predominantly secreted by immune cells and tumor-associated stromal cells, cytokines modulate target cell proliferation and differentiation through transmembrane receptor-mediated signaling pathways (69).

For instance, IFNγ, a Type II interferon produced by Th1 and CD8+ T cells, plays a pivotal role in anti-tumor and immunoregulatory processes. IFNγ activates the JAK/STAT signaling pathway to regulate the transcription of downstream genes involved in antigen presentation and chemokine expression. Mariathasan et al. observed a significant upregulation of IFNγ expression among responders to anti-PD-L1 therapy (56). Conversely, transforming growth factor-β (TGF-β) is frequently associated with poor prognosis, as it promotes tumor growth by driving fibroblast activation, angiogenesis, immunosuppression, and epithelial-mesenchymal transition (EMT) (70). In the IMvigor210 trial, high expression of TGFB1 and TGFBR2 was linked to anti-PD-L1 resistance and diminished OS (56).

Finally, chemokines—secreted by fibroblasts, dendritic cells, macrophages, and malignant cells—act as the primary drivers of cell trafficking. Depending on the specific cell types they recruit, chemokines can exert either pro- or anti-tumorigenic effects. In particular, CXCL9 and CXCL10 have been identified as key correlates of PD-L1 positivity and positive responses to PD-1/PD-L1 inhibition (9, 37, 56). Results from the Checkmate 275 trial further confirmed that responders with mUC exhibit significantly elevated expression levels of CXCL9 and CXCL10.

## 3 Clinical biomarkers for predicting response to PD-1/PD-L1 inhibition

### 3.1 PD-L1 expression

Programmed death-ligand 1 (PD-L1) serves as a critical immune checkpoint molecule essential for maintaining immunological homeostasis. While PD-L1 is moderately expressed in various normal tissues under physiological conditions, the tumor microenvironment exploits this pathway to evade immune surveillance. Specifically, the interaction between PD-1 and PD-L1 transmits inhibitory signals that diminish T-cell activity (71).

Rather than functioning as a static biomarker, PD-L1 expression is subject to rigorous dynamic regulation through various RNA modifications and post-translational modifications (PTMs) (72, 73). The current limitations of PD-L1 as an independent predictive indicator are largely rooted in these complex regulatory networks.

#### 3.1.1 Hierarchical evolution of expression regulation: from mRNA fate to protein shielding

Functional expression of PD-L1 begins with the precise modulation of mRNA stability. In the bladder cancer microenvironment, inflammatory signaling activates the JNK-c-Jun axis, which subsequently upregulates the methyltransferase METTL3 (72). METTL3 catalyzes N^6^-methyladenosine (m^6^A) modification within the 3’-UTR of PD-L1 mRNA. This modification is recognized by reader proteins, significantly enhancing mRNA stability and promoting translation (72). Furthermore, YTHDF1 facilitates protein synthesis by strengthening the eIF5B-PD-L1 axis (73). This mechanism elucidates why certain patients maintain high basal PD-L1 levels even in the absence of typical inflammatory stimuli.

Once translation is complete, the stability and “visibility” of PD-L1 depend on the interplay of various PTMs. Phosphorylation of PD-L1 at Y112, induced by the IL-6/JAK1 axis, functions as a molecular scaffold to recruit the glycosyltransferase STT3A (74). This recruitment initiates N-glycosylation at multiple sites (including N35, N192, N200, and N219), creating a “protective shield” that prevents proteasomal degradation (75, 76). Additionally, NEK2 reinforces protein stability by phosphorylating T194/T210 residues (76), while TMUB1 drives tumor immune evasion by upregulating STT3A to promote N-glycosylation (77). Simultaneously, palmitoylation serves as another critical regulator of PD-L1 membrane stability.

#### 3.1.2 PTM-driven “Clinical paradox”: epitope masking and diagnostic reliability

N-glycosylation (gPD-L1) does more than regulate protein stability; it creates a significant pitfall in clinical diagnostics known as epitope masking.

Diagnostic Distortion and False Negatives: Bulky glycan chains physically obstruct the recognition of PD-L1 extracellular domain epitopes by commonly used clinical antibodies, such as clones 22C3, SP263, and SP142 (78). Because the antigenic epitopes targeted by these clones are often shielded by highly glycosylated regions, numerous tumor cells with functional PD-L1 expression are erroneously classified as “false negatives” in routine immunohistochemistry (IHC) assays (79). This phenomenon elucidates why a subset of patients clinically staged as PD-L1 negative still derive significant benefit from immunotherapy.

The Biological Double-Edged Sword: Glycosylation, particularly N-linked glycosylation, exerts a steric hindrance effect that effectively blocks GSK3β-mediated phosphorylation and subsequent ubiquitination-dependent degradation (80). By shielding the protein from these degradative pathways, glycosylation significantly extends the half-life of PD-L1 and enhances its stability within the tumor microenvironment (81, 82).

Diagnostic Optimization Strategies: To address the interference caused by glycosylation, translational research has validated the use of PNGase F to deglycosylate samples prior to IHC staining. This enzymatic pretreatment removes glycan chains and re-exposes masked epitopes (83). Implementing deglycosylation protocols significantly improves the correlation between PD-L1 detection results and clinical outcomes, offering a robust framework for more precise patient stratification.

#### 3.1.3 Subtype-specific turnover: the FGFR3 axis and protein degradation

The dynamic degradation of PD-L1 underpins the distinct immunological profiles observed across various molecular subtypes of bladder cancer. In luminal-papillary tumors, the activation of oncogenic FGFR3 leads to the phosphorylation of the E3 ubiquitin ligase NEDD4. Once activated, NEDD4 acts in synergy with other ligases, such as RNF144A, to directly mediate the K48-linked polyubiquitination of PD-L1. This modification targets the protein for proteasomal degradation, thereby enhancing CD8+ T-cell antitumoral activity and contributing to a “cold” immune landscape (40). Conversely, the deubiquitinase USP7 stabilizes PD-L1 through direct physical interaction, preventing its degradation and maintaining the immunosuppressive state of the tumor (84).

#### 3.1.4 Predictive value and challenges of clinical biomarkers

In clinical settings, PD-L1 expression on tumor cells (TCs) and tumor-infiltrating immune cells (ICs) is typically evaluated via immunohistochemistry (IHC) to stratify patient cohorts. Several studies indicate that high PD-L1 expression correlates with superior therapeutic responses. For example, exploratory analysis from the IMvigor130 study demonstrated significant efficacy of atezolizumab monotherapy in patients with high PD-L1 expression (85). Similarly, the Checkmate 274 trial showed that adjuvant nivolumab significantly prolonged disease-free survival (DFS) compared to placebo in high-risk MIBC patients, both in the intent-to-treat population and in those with PD-L1 expression ≥ 1% (86).

Despite these findings, the reliability of PD-L1 as a standalone predictive biomarker remains contested (71). In the IMvigor210 trial, no significant differences in ORRs were observed across cohorts with varying PD-L1 immune cell scores (87). Furthermore, the IMvigor211 trial reported that atezolizumab failed to significantly extend OS compared to chemotherapy in patients with platinum-refractory mUC overexpressing PD-L1 (88). These discrepancies may stem from the dynamic heterogeneity of PD-L1 expression within the TME and the current lack of standardized analytical platforms for PD-L1 assessment.

### 3.2 Tumor mutational burden

TMB is a quantitative metric defined as the total number of somatic non-synonymous mutations per megabase (mut/Mb) within the tumor genome. Bladder cancer typically exhibits intermediate-to-high TMB levels compared to other malignancies (89, 90), a phenomenon likely driven by environmental mutagens—such as tobacco smoke—that induce significant genomic instability. High TMB is associated with an increased diversity and load of neoantigens, which serve as primary triggers for the recruitment and activation of CTLs (91, 92). This enhanced immunogenicity significantly improves clinical response rates to immunotherapy.

Subgroup analyses of the IMvigor210 cohort II, utilizing sequencing of 315 cancer-driver genes, demonstrated that responders to anti-PD-L1 therapy possessed significantly higher TMB than non-responders (37). Furthermore, TMB is a robust prognostic indicator in bladder cancer; data from IMvigor210 Cohort 1 showed that patients with elevated TMB achieved prolonged OS (87). Similarly, clinical trials involving pembrolizumab monotherapy (KEYNOTE-052 and KEYNOTE-045) and nivolumab (CheckMate 275) confirmed a positive correlation between TMB and OS (93–95). This correlation suggests that a high TMB state enhances immune surveillance, enabling the immune system to identify and eliminate residual malignant cells more effectively, thereby reducing the risk of recurrence. Consequently, TMB is a promising biomarker for predicting ICI efficacy (90, 96, 97). Patients with high TMB may benefit from immunotherapy as a primary intervention, whereas those with low TMB might require alternative or combinatorial strategies involving surgery, chemotherapy, or targeted therapy.

The clinical application of TMB continues to evolve. While whole-exome sequencing (WES) was initially the gold standard for TMB assessment, its high cost and technical complexity have led to the adoption of next-generation sequencing (NGS) panels as a pragmatic clinical alternative (98, 99). However, TMB quantification is susceptible to significant biases arising from differences in sequencing platforms, panel sizes, and bioinformatic algorithms.

Furthermore, the optimal TMB threshold for different cancer types remains a subject of investigation. Although the FDA granted accelerated approval in 2020 for pembrolizumab in unresectable or metastatic solid tumors with a threshold of ≥ 10 mut/Mb, some studies suggest that for highly mutated cancers—including melanoma, colorectal, bladder, and non-small cell lung cancer—a more stringent cutoff of ≥13 mut/Mb may be more appropriate (90). Therefore, the development of standardized and precise analytical workflows and interpretive criteria is essential for the routine clinical implementation of TMB.

### 3.3 Circulating tumor DNA

Circulating tumor DNA (ctDNA) refers to fragmented DNA shed into the bloodstream by malignant cells. The detection and quantification of plasma ctDNA have emerged as robust, non-invasive modalities (100). They are utilized for bladder cancer screening, early diagnosis, minimal residual disease (MRD) detection, prognostic evaluation, and real-time monitoring of therapeutic efficacy (101–103). Substantial evidence indicates that ctDNA analysis offers superior sensitivity compared to conventional imaging techniques for the early detection of tumor recurrence. Driven by recent high-impact clinical trials, the role of ctDNA has fundamentally shifted. It is no longer merely a prognostic biomarker but has become a predictive tool that directly guides perioperative immunotherapy decisions.

Recent studies highlight a significant correlation between dynamic fluctuations in ctDNA levels and responsiveness to immunotherapy. In the post-surgical setting, ctDNA positivity is highly indicative of molecular relapse. Retrospective analyses of the IMvigor010 trial initially suggested that ctDNA positivity could serve as a potential biomarker to identify patients suitable for adjuvant atezolizumab following cystectomy. Furthermore, patients with persistent ctDNA positivity during immunotherapy exhibited a markedly higher risk of recurrence and disease progression (104). Building upon these exploratory findings, the landmark Phase III IMvigor011 trial prospectively validated a ctDNA-guided treatment strategy (105). In this study, the administration of adjuvant atezolizumab was guided exclusively by ctDNA positivity during postoperative surveillance. Results demonstrated that, compared to placebo, the atezolizumab cohort experienced a significant 41% reduction in the risk of death (OS HR = 0.59). Additionally, the risk of disease recurrence or death was reduced by 36% (disease-free survival [DFS] HR = 0.64). Crucially, patients who remained persistently ctDNA-negative achieved an exceptional 1-year DFS rate of 95% without receiving any adjuvant therapy. This provides Level 1 evidence that ctDNA-negative patients can safely forgo systemic treatment, thereby avoiding unnecessary toxicity and overtreatment (treatment de-escalation). Similarly, the 5-year extended follow-up of the CheckMate 274 trial further reinforced the predictive value of ctDNA. Patients with detectable baseline ctDNA derived a substantial DFS benefit from adjuvant nivolumab (HR = 0.35). Conversely, patients with undetectable ctDNA received no additional DFS benefit compared to placebo (HR = 0.99). This clearly underscores the utility of ctDNA for patient stratification and precision therapy (106).

Beyond the adjuvant setting, dynamic ctDNA clearance is increasingly recognized as an early indicator of therapeutic success. The TOMBOLA trial represents the first study to utilize serial ctDNA measurements to guide clinical decision-making in bladder cancer. Results revealed significant differences in OS and recurrence-free survival (RFS) between ctDNA-positive and ctDNA-negative cohorts (107). The study further demonstrated a median lead time of 90 days from ctDNA detection to radiographically confirmed recurrence. More importantly, the immediate initiation of atezolizumab upon molecular relapse (ctDNA detection) achieved a 60% complete response rate, highlighting a critical window for “molecular salvage.” In the broader perioperative setting, trials such as NIAGARA have further established that combining immunotherapy, like durvalumab, with neoadjuvant chemotherapy can significantly enhance ctDNA clearance rates. This improvement subsequently translates into extended event-free survival (EFS) and enhanced overall clinical outcomes.

Despite these paradigm-shifting advancements, the comprehensive clinical implementation of ctDNA testing continues to face several technical hurdles. Because the concentration of ctDNA in plasma is typically low, highly sensitive detection methods are required. Examples include droplet digital PCR (ddPCR) and next-generation sequencing (NGS) (108). Furthermore, different detection strategies possess inherent limitations. Tumor-informed approaches require available tumor tissue and involve longer turnaround times. Conversely, tumor-agnostic methods necessitate the careful filtration of background noise caused by clonal hematopoiesis (CHIP) (109, 110). Additionally, these technologies are not yet universally accessible, as they are often constrained by high costs and computational complexity (111). Looking ahead, detection sensitivity will continue to improve and integrate with large-scale clinical data analytics. Next-generation adaptive trials, such as the MODERN study (NCT05987241), will further elucidate the safety of ctDNA-guided treatment de-escalation, as well as the efficacy of intensified regimens for ctDNA-positive patients. Consequently, this will solidify the central role of ctDNA in optimizing PD-1/PD-L1 inhibitor therapy for bladder cancer and advancing precision oncology.

### 3.4 Additional biomarkers

Beyond established genomic indicators, several emerging molecular and systemic factors contribute to the complexity of the TME and influence therapeutic outcomes. The overexpression of CD73 or CD39 catalyzes the extracellular accumulation of adenosine, a potent immunosuppressive metabolite that inhibits T-cell activity and significantly attenuates anti-tumor immunity (112).

Additionally, the presence of soluble immunosuppressive factors, such as interleukin-8 (IL-8) and vascular endothelial growth factor (VEGF), exacerbates the formation of an exhausted immune milieu, which is frequently associated with poor clinical prognosis (113). Systemic factors also play a critical role; for instance, dysbiosis of the gut microbiota disrupts the homeostatic “gut-immune axis,” further modulating the efficacy of ICIs (114).

Future investigations into these diverse biomarkers—ranging from metabolic pathways to the systemic microbiome—are essential for developing multifaceted predictive models and identifying novel therapeutic targets.

## 4 Synergistic combinatorial strategies for PD-1/PD-L1 inhibition

### 4.1 Combination with chemotherapy

Chemotherapeutic agents enhance the immunogenicity of malignant cells and modulate the composition and functionality of immune populations within the TME. Specifically, platinum-based agents—standard in bladder cancer care—induce DNA damage and trigger immunogenic cell death (ICD). This process facilitates the phagocytosis of apoptotic debris by APCs, which subsequently present tumor-derived neoantigens to the adaptive immune system (115, 116).

Preclinical studies utilizing MB49 and MBT-2 murine bladder cancer models have demonstrated that combining anti-PD-L1 therapy with cisplatin yields superior therapeutic efficacy compared to chemotherapy alone (117, 118). Despite these promising laboratory results, clinical trials investigating concurrent PD-1/PD-L1 inhibition and chemotherapy have yielded divergent outcomes. The KEYNOTE-361 and IMvigor130 trials failed to demonstrate a significant clinical benefit for combination therapy or ICI monotherapy over standard platinum-based chemotherapy in patients with mUC (119, 120).

In contrast, the 2023 CheckMate 901 trial reported that the addition of nivolumab to gemcitabine-cisplatin significantly prolonged both PFS and OS (121). This clinical success has shifted research attention toward the impact of treatment sequencing, where sequential administration may be pivotal. In this framework, chemotherapy is utilized to “prime” the TME, establishing an immune-favorable state before PD-1/PD-L1 inhibitors are introduced to amplify the anti-tumor response (122).

The success of the JAVELIN Bladder 100 trial underscored the importance of treatment sequencing (6). While concurrent ICI-chemotherapy combinations in trials like KEYNOTE-361 and IMvigor130 did not reach their primary endpoints, the sequential introduction of avelumab to ‘prime’ the immune environment after platinum-induced immunogenic cell death (ICD) significantly extended median OS to 21.4 months compared to 14.3 months with best supportive care alone (HR = 0.69). This paradigm emphasizes that avelumab’s role is not a substitute for chemotherapy in cisplatin-ineligible patients, but a consolidation strategy for those who benefit from any platinum-containing first-line induction. Similarly, the CheckMate 274 trial showed that adjuvant nivolumab improved outcomes in patients who had previously received neoadjuvant cisplatin, further supporting the validity of sequential protocols (86). Future investigations must now focus on optimizing these sequential regimens and identifying the precise therapeutic windows to maximize clinical benefit.

### 4.2 Combination with radiotherapy

Ionizing radiation triggers genomic DNA double-strand breaks and generates reactive oxygen species (ROS) through direct energy deposition, leading to cell cycle arrest, apoptosis, and cell death (123). Beyond direct cytotoxicity, radiotherapy induces immunogenic effects within the TME by stimulating the release of IFNγ. Furthermore, it strengthens the adaptive immune system by upregulating MHC-I expression, which enhances the activity of CTLs.

The integration of radiotherapy with PD-1/PD-L1 inhibitors achieves synergistic anti-tumor effects through antigen-presenting cell (APC)-mediated cross-presentation and subsequent T-cell activation (124, 125). The PLUMMB clinical trial (NCT02560636) was initiated to evaluate the safety and efficacy of pembrolizumab combined with radiotherapy in patients with MIBC or locally advanced disease (126). However, this trial was prematurely terminated due to the emergence of severe toxicities (126).

To date, no clinical trials investigating the combination of radiotherapy and PD-1/PD-L1 inhibition in bladder cancer have reached completion, although several studies remain active. These include a Phase II trial of nivolumab plus radiotherapy (NCT03529890) and a multi-arm Phase I/II study evaluating durvalumab in combination with Bacillus Calmette-Guérin (BCG) and radiotherapy (NCT03317158). Notably, variables such as the fractionation schedule, cumulative dosage, and the specific target volume are critical factors that dictate the synergy between radiotherapy and subsequent immunotherapy.

### 4.3 Combination with targeted therapies

DNA Damage Response (DDR) Inhibitors: Current therapeutic strategies leveraging DDR deficiencies primarily focus on targeted agents that disrupt molecular pathways involved in DNA repair. Poly (ADP-ribose) polymerase (PARP) inhibitors, for instance, interfere with the repair of single-strand breaks, inducing synthetic lethality in malignancies harboring germline or somatic BRCA1/2 mutations (127). Beyond direct cytotoxicity, PARP inhibition promotes genomic instability, leading to the accumulation of somatic mutations and a significant elevation in TMB. This process subsequently increases neoantigen load and enhances tumor immunogenicity (128, 129).

Preclinical evidence suggests that PARP inhibition upregulates PD-L1 expression, creating a synergistic anti-tumor environment when combined with PD-1/PD-L1 blockade (130). In MIBC, the combination of the PARP inhibitor olaparib with anti-PD-L1 therapy has been shown to increase the expression of chemokines, TNF-α, VEGF, and IFN-γ, while concurrently boosting TIL density (130). Notably, clinical studies have reported a pathologic complete response (pCR) rate of 44.5% in patients treated with this combinatorial approach (131).

Antibody-Drug Conjugates (ADCs): Nectin-4 is highly expressed in urothelial carcinoma cells, providing a selective target for Enfortumab Vedotin (EV). EV is an ADC composed of a human IgG1 monoclonal antibody targeting Nectin-4, conjugated to the cytotoxic payload monomethyl auristatin E (MMAE). Upon internalization, EV releases MMAE to induce cell cycle arrest and apoptosis (132, 133). Crucially, EV also triggers ICD, which facilitates the recruitment and activation of T cells (134, 135).

When integrated with PD-1/PD-L1 inhibitors, EV exhibits potent synergistic anti-tumor activity (136–138). Results from the landmark EV-302 trial demonstrated that first-line therapy with EV plus pembrolizumab significantly improved OS and PFS in patients with locally advanced or mUC (139). This regimen represents the first combination in three decades to demonstrate superiority over platinum-based chemotherapy, effectively establishing a new standard of care for advanced bladder cancer.

Beyond established protocols, VEGF inhibitors facilitate therapeutic efficacy by promoting T-cell recruitment into the tumor parenchyma and alleviating immunosuppression through the depletion of regulatory T cells (Tregs), thereby sensitizing the TME to PD-1/PD-L1 blockade.

While this synergy is well-documented across various malignancies, clinical evidence for localized bladder cancer remains in the nascent stages. Other emerging strategies include the inhibition of discoidin domain receptor 2 (DDR2); specifically, DDR2 depletion has been shown to enhance anti-PD-1 sensitivity *in vivo*, and the combination of the tyrosine kinase inhibitor dasatinib with anti-PD-1 therapy significantly augments CD8+ T-cell infiltration (14). Furthermore, the TGF-β receptor inhibitor vactosertib is currently under evaluation in combination with durvalumab for refractory or recurrent disease within a Phase II clinical framework (NCT04064190) (140).

Simultaneously, investigations into cell cycle control are progressing, with trials assessing the utility of CDK4/6 inhibitors—such as trilaciclib (NCT04887831) and palbociclib (NCT06364956)—to overcome resistance to standard platinum-based regimens. Ultimately, ongoing research into additional molecular vulnerabilities, including HER2 and FGFR, aims to refine personalized combinatorial protocols and maximize ORRs for PD-1/PD-L1-directed therapies.

### 4.4 Combination with nanomedicine

The synergistic integration of various nanomedicines with immunotherapy offers a potent strategy to reverse the immunosuppressive TME, facilitating the conversion of immunologically “cold” tumors into “hot” phenotypes. These nanoplatforms typically operate through three primary targeting pathways: the malignant cells, the TME, and the peripheral immune system (141).

Specifically, tumor-targeted nanomedicines are designed to induce ICD and bolster the tumor-immune cycle (141, 142). When directed at the TME, these agents can suppress inhibitory populations—such as myeloid-derived suppressor cells (MDSCs) and M2-polarized TAMs—and neutralize immunosuppressive molecules like TGFβ and adenosine (ADO). Simultaneously, they augment the activity and functionality of immune effectors, including macrophages and CTLs (143–145). Alternatively, nanomedicines targeting the peripheral immune system aim to catalyze antigen presentation and systemic CTL production (146, 147).

Recent studies in bladder cancer have begun to explore these nanotherapeutic integrations with PD-1/PD-L1 inhibitors. For instance, Zhou et al. utilized macrophage-derived exosome-mimicking nanovesicles (EMVs) to co-deliver the CD73 inhibitor (AB680) and a PD-L1 antagonist. This nanocomplex (AB680@EMVs-aPDL1) demonstrated superior tumor targeting and significantly promoted CTL activation and infiltration in murine models (148). Furthermore, combining porphyrin-based PLZ4-nanoparticles (PNP) with photodynamic therapy (PDT) and anti-PD-1 blockade significantly improved median survival in SV40 T/Ras dual-transgenic mice harboring spontaneous bladder cancer (149).

Despite this progress, the clinical translation of nanomedicine-ICI combinations faces substantial hurdles. The inherent structural complexity and diversity of nanoplatforms may lead to unpredictable immune responses, necessitating more profound investigations into their specific immunomodulatory mechanisms. Furthermore, ensuring the long-term safety and consistent therapeutic efficacy of these formulations remains a critical prerequisite for future clinical application.

### 4.5 Alternative and emerging modalities

Beyond conventional combinations, several emerging therapeutic strategies are being explored to augment the anti-tumor response. Cellular therapies, including chimeric antigen receptor (CAR)-T cell and TIL therapies, empower the immune response by enriching the repertoire of tumor-specific effector T cells.

Similarly, cancer vaccines facilitate therapeutic efficacy by stimulating the expansion of antigen-specific T-cell populations targeted against malignancy-associated epitopes (150). Oncolytic viral therapy serves a dual role: it effectuates direct tumor lysis while simultaneously triggering the release of sequestered tumor antigens and activating innate immune signaling pathways, thereby reinforcing systemic anti-tumor immunity (151).

**Figure 2:**
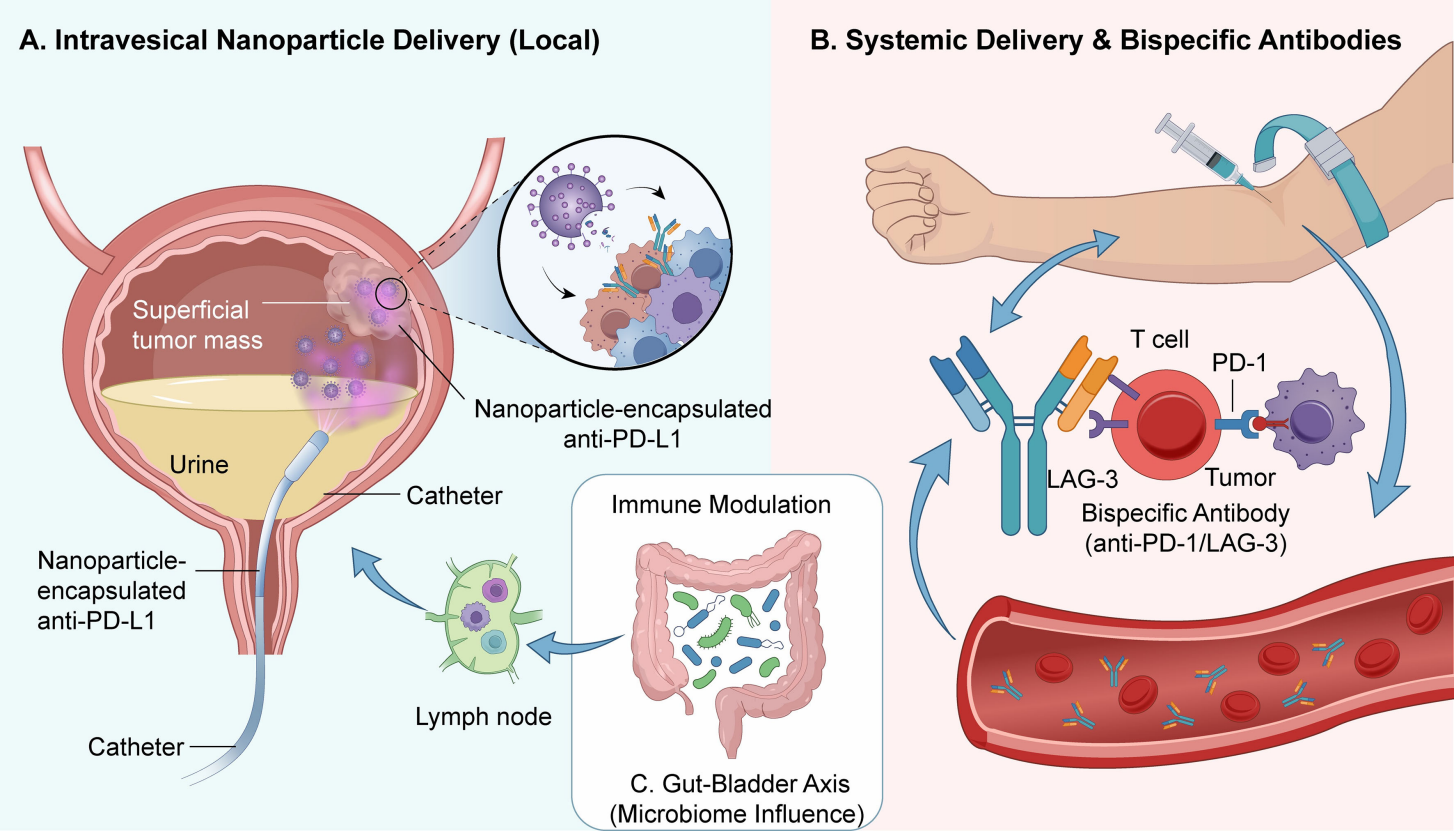
Novel approaches involving innovative delivery systems, emerging therapeutic targets, and microbiome modulation to enhance bladder cancer immunotherapy. A tripartite schematic illustrating futuristic strategies designed to improve the efficacy and specificity of PD-1/PD-L1 blockade in bladder cancer. **(A)** Intravesical Nanoparticle Delivery (Local): A diagram of the bladder demonstrating catheter-mediated intravesical instillation. Nanoparticle-encapsulated anti-PD-L1 antibodies are delivered directly into the bladder lumen. These nanocarriers are engineered to enhance penetration into the urothelium and specifically accumulate within superficial tumor masses. The magnified inset shows the targeted, sustained release of therapeutic antibodies from the degrading nanoparticles directly at the tumor site, ensuring high local drug concentration while minimizing systemic exposure and toxicity. **(B)** Systemic Delivery & Bispecific Antibodies: An illustration depicting the systemic intravenous administration of next-generation immunotherapeutics. The central mechanism highlights a bispecific antibody designed to simultaneously engage two distinct co-inhibitory receptors: PD-1 and LAG-3 (Lymphocyte-activation gene 3) on the surface of an exhausted T cell. By achieving dual blockade of these checkpoints, the bispecific antibody synergistically reinvigorates T cell function and enhances cytotoxic activity against tumor cells. The blood vessel cross-section indicates systemic circulation to reach both primary and metastatic sites. **(C)** Gut-Bladder Axis (Microbiome Influence): A conceptual model illustrating the “gut-bladder axis” and the immunomodulatory role of the gut microbiome. Metabolites and signals derived from diverse intestinal bacteria influence the training and activation of immune cells within gut-associated lymph nodes. These modulated immune cells subsequently migrate and shape the immune landscape within the bladder microenvironment, distant from the gut, thereby impacting the responsiveness to immunotherapy.

Cytokine-based interventions, particularly those involving IL-2 and IL-15, are utilized to drive the proliferation and activation of T and NK cells, intensifying the immune assault on malignant tissues (152). Furthermore, systemic modulation of the gut microbiota—via probiotic or prebiotic supplementation and FMT—has emerged as a viable strategy to reshape the immune microenvironment and enhance the clinical responsiveness to PD-1/PD-L1 inhibition (153) (**Figure 2**).

## 5 Conclusion and future perspectives

The clinical integration of PD -1/PD - L1 inhibitors has fundamentally revolutionized the therapeutic paradigm for bladder cancer. However, significant inter-individual variability in treatment response and the inevitable emergence of acquired resistance remain formidable challenges that necessitate urgent resolution. Future research must transcend the search for isolated biomarkers or the arbitrary combination of existing therapies; instead, it must evolve toward a more precise, dynamic, and multi-targeted synergistic framework.

### 5.1 Dynamic multi-omics and precision monitoring

Traditional single-point biopsies are inherently limited in their ability to capture the spatiotemporal evolution of tumor heterogeneity. Future strategies should prioritize a closed-loop system of multi-omics dynamic monitoring. By integrating genomic features such as Loss of Y (LOY) status, ctDNA kinetics, and single-cell derived tertiary lymphoid structure (TLS) signatures, researchers can develop real-time risk assessment models. These models will allow therapeutic decisions to be recalibrated in tandem with the evolving TME, facilitating truly individualized precision medicine.

### 5.2 Next-generation post-translational modulators

Next-generation immunomodulators targeting PTMs represent a nascent yet high-potential frontier. Developing small-molecule inhibitors that specifically regulate PD-L1 stability—by modulating its glycosylation, ubiquitination, or phosphorylation—offers a strategic advantage. These agents may enhance immunotherapeutic sensitivity at the protein level and effectively bypass the selective pressures and resistance mechanisms associated with conventional monoclonal antibody therapies.

### 5.3 Integration of cellular therapy and novel checkpoints

A pivotal trend in the field is the consolidation of cellular therapies with novel checkpoint blockades. Beyond expanding our understanding of established checkpoints such as CTLA-4, LAG-3, and TIGIT, the preliminary application of CAR-T and CAR-NK therapies in bladder cancer signals a fundamental shift in treatment philosophy. The objective is moving from merely “disinhibiting” immune suppression toward a “proactive” and multi-targeted offensive strategy.

## 6 Summary

By deeply interrogating the unique biological hallmarks of bladder cancer and precisely reshaping the TME through multimodal strategies, the management of advanced urothelial carcinoma is poised for a total metamorphosis. The ultimate goal is to transition this malignancy into a “long-term, manageable” chronic disease paradigm, providing patients with significantly improved survival rates and a superior quality of life.
